# Investigating the effects of dietary and management modifications on *Salmonella enterica* population in harvest-ready beef cattle

**DOI:** 10.1128/spectrum.00264-25

**Published:** 2025-07-11

**Authors:** Yesica Botero, Kasi Schneid, Kendall L. Samuelson, John T. Richeson, Ty E. Lawrence, Gizem Levent

**Affiliations:** 1 Texas A&M University Department of Veterinary Integrative Biosciences, College Station, Texas, USA; 2 Texas Tech University School of Veterinary Medicine, Amarillo, Texas, USA; 3 West Texas A&M University Department of Agricultural Sciences, Canyon, Texas, USA; University of Minnesota Twin Cities, Minneapolis, Minnesota, USA

**Keywords:** cattle management, diet, management, *Salmonella*

## Abstract

*Salmonella enterica*, a foodborne pathogen, poses a significant public health risk, particularly because of multidrug-resistant strains. Cattle are one of the known reservoirs of *Salmonella* that contaminates beef products. Whereas antibiotic use in cattle contributes to the selection of antibiotic-resistant strains, it is essential to understand other factors that may influence *Salmonella* dynamics in cattle and their feedlot environment to mitigate and control related public health risks effectively. With this double-blinded, randomized controlled feedlot study, originally designed to evaluate the effects of dietary (high-starch vs. low-starch) and feeding management (erratic vs. regular feeding) changes on animal performance and liver abscess formation, we aimed to investigate *Salmonella* prevalence and antibiotic susceptibility profiles in cattle and the feedlot during the approximately 222-day-long feeding period and at harvest. Fecal, hide, lymph node, and soil samples were collected from study cattle, resulting in 863 fecal samples, 309 hide swabs, 131 lymph nodes, and 288 composite pen samples. *Salmonella* was isolated using standard methods involving non-selective and selective media. Among the 536 isolates tested for phenotypic antibiotic susceptibility, two soil-origin isolates recovered during the feeding period were antibiotic-resistant and likely did not remain persistent due to a lack of selective pressure. Overall, treatments did not affect (*P* > 0.05) *Salmonella* prevalence in cattle feces, hides, or the feedlot environment. However, the high-starch diet demonstrated potential as a pre-harvest intervention, reducing *Salmonella* prevalence by 0.20 (95% *CIs* 0.02–0.43) in cattle lymph nodes, which may help mitigate and control *Salmonella* risks in beef products.

**IMPORTANCE:**

*Salmonella* is a leading foodborne pathogen, causing numerous infections, hospitalizations, and deaths annually in the United States. Cattle are known to harbor *Salmonella*; therefore, understanding factors influencing *Salmonella* dynamics in cattle and the feedlot environment is essential to mitigate or control associated risks in beef products. Here, we investigated the effects of dietary energy (high-starch vs. low-starch diet) and feeding management (regular vs. erratic feeding) on *Salmonella* in cattle and the feedlot environment. Our findings suggest that a high-starch diet may have the potential to reduce *Salmonella* prevalence in cattle lymph nodes, a potential source of *Salmonella* outbreaks. In addition, most *Salmonella* isolates were pansusceptible, with only a few antibiotic-resistant strains identified, which were likely not persistent due to a lack of selective pressure. Further research is needed to evaluate the use of a high-starch diet as a pre-harvest intervention to control and mitigate *Salmonella* risks in final beef products.

## INTRODUCTION

Nontyphoidal *Salmonella* is considered one of the leading foodborne pathogens in the United States (1), causing an estimated 1.35 million infections, 26,500 hospitalizations, and 420 deaths annually (dx.doi.org/10.15620/cdc:82532 [Accessed June 2025]) (2). The Centers for Disease Control and Prevention (CDC) considers drug-resistant *Salmonella* a serious public health threat because of rising resistance to key antibiotics, including ciprofloxacin, azithromycin, and ceftriaxone used for the treatment of severe salmonellosis in humans (2, 3). In recent years, an increasing number of human clinical isolates have shown resistance—or reduced susceptibility—to these antibiotics used for *Salmonella* treatment (4).

Foodborne *Salmonella* outbreaks continue to pose a significant public health concern. According to the Interagency Food Safety Analytics Collaboration (IFSAC) 2021 report published in 2023, a total of 987 foodborne *Salmonella* human outbreaks were described, of which 55.4% were attributed to contaminated animal-origin food products and 44.6% to other contaminated products such as fruits, seeded vegetables, and grains. Among the top animal-origin food products, the estimated attribution of outbreaks was 18.6% for chicken, 12.1% for pork, and 6.5% for beef (stacks.cdc.gov/view/cdc/138757 [Accessed June 2025]) (5).

Although there are more than 2,500 *Salmonella* serotypes identified to date, each characterized by three major distinct antigens (O, H—phase 1, and H—phase 2 antigens) according to the Kaufmann-White serotyping scheme (6), only a few are responsible for human foodborne outbreaks (stacks.cdc.gov/view/cdc/22161 [Accessed June 2025]) (7). Certain serotypes, including Enteritidis, Newport, Typhimurium, and Montevideo, are frequently associated with human outbreaks and hospitalizations (stacks.cdc.gov/view/cdc/58450 [Accessed June 2025]) (8) and are occasionally detected in beef products (www.fsis.usda.gov/sites/default/files/media_file/documents/Dataset_QSR_Salmonella_PercentPositive_FY2024Q3.xlsx [Accessed June 2025]) (9, 10). Recently, serotypes Saint Paul, Dublin, and Newport have also been linked to ground beef-related human outbreaks (archive.cdc.gov/#/details?url=https://www.cdc.gov/salmonella/newport-10-18/index.html, archive.cdc.gov/#/details?archive_url=https://archive.cdc.gov/www_cdc_gov/salmonella/dublin-11-19/index.html, www.cdc.gov/salmonella/outbreaks/saintpaul-07-23/investigation.html [Accessed June 2025]) (11–13). These serotypes, which are important for public health, can also be detected in cattle and their environment (www.fsis.usda.gov/sites/default/files/media_file/2020-07/Beef-Veal-Carcass-Baseline-Study-Report.pdf [Accessed June 2025]) (14–17).

Even though cattle are asymptomatic carriers of *Salmonella,* they can still contaminate the feedlot environment via feces (14, 15, 18). Cattle are known reservoirs for *Salmonella*, and the epidemiology of *Salmonella* in cattle and the feedlot environment is complex. One primary route of infection is fecal-oral transmission, often facilitated by cattle’s grooming behavior, as hide surfaces can become contaminated with feces (19, 20). In addition, cattle can be exposed to *Salmonella* and potentially infected through contaminated water or feed (21, 22).

Once cattle become carriers of *Salmonella*, beef and beef products can also be contaminated. The contamination of beef carcasses with *Salmonella* can occur via cattle feces or hide during hide removal and evisceration (17), or indirectly through the abattoir environment and equipment used during the harvesting process (23, 24). In addition to carcass contamination, final beef products can also become contaminated with *Salmonella*. For example, ground beef, a source of beef-related *Salmonella* outbreaks (11–13), can be contaminated through *Salmonella* found in cattle lymph nodes. These lymph nodes are often embedded in fat tissue, which is important for ground beef production. During the fat-trimming process, *Salmonella*-harboring lymph nodes can be incorporated into the final beef products (16, 25, 26).

In addition to the cattle feces, hide, and lymph nodes, *Salmonella* has been detected in liver abscesses of cattle (27). Although livers with abscesses are unfit for human consumption and condemned, liver abscesses pose animal health and performance concerns, coupled with logistical challenges during harvest. Liver abscesses in cattle are often linked to intense feeding practices, such as feeding cattle a high proportion of grain containing rapidly available starch. This practice can lead to acute ruminal acidosis (28), which causes rumen lesions that allow bacteria—such as *Fusobacterium necrophorum* and *Trueperella pyogenes*, the primary causative agents of liver abscesses, or other bacteria, such as *Salmonella*—to breach the rumen wall and enter the hepatic portal circulation (29, 30).

To reduce the public health risks of *Salmonella* in beef products, several post-harvest interventions are applied at the abattoir level, including organic acid, oxidizer, thermal, and other non-thermal treatments (31). Interventions such as steam vacuuming, lactic acid, acetic acid, and ozonated water are routinely used on hide and/or trimming, and lactic acid, citric acid, peroxyacetic, and acidified sodium chlorite are used on carcass surfaces to reduce *Salmonella* load (31–33). These interventions are effective at decontaminating surfaces; however, they do not reduce the *Salmonella* load in ground beef products, which often originates from *Salmonella* present in the lymph nodes (31). Therefore, effective pre-harvest interventions targeting *Salmonella* in cattle, particularly within their lymph nodes, are essential to reduce the incidence of ground-beef origin *Salmonella* outbreaks.

To develop an effective pre-harvest intervention, it is crucial to understand the risk factors associated with *Salmonella* in feedlots. *Salmonella* shedding and exposure can vary and are influenced by external factors, including seasonal weather changes, feedlot environment, feedlot management practices, diet, stress, and cattle source, as well as host-related factors such as behavior and age (20, 34, 35). *Salmonella* in feedlots is highly clonal in cattle, with these clones detected in the environment, on hides, in feces, and in lymph nodes, which can be influenced by the length of housing and pen design (16, 36, 37).

Several pre-harvest interventions are suggested to be effective in reducing the *Salmonella* load in cattle. A subset of these interventions involves dietary changes such as direct-fed microbials (DFMs) (38–43) or other dietary modifications, including silage, citrus pulp, urea, brewer’s grain, corn gluten feed, or cottonseed (44–49), while others may target immunological protection through vaccination (50, 51) or the environmental changes through bacteriophage applications (52). Among those, dietary changes may be particularly promising as they can help reduce *Salmonella* colonization in the gastrointestinal tract (GIT) and potentially in the lymph nodes during the feeding period. Alterations in cattle diet may also influence microenvironmental GIT conditions, which, in turn, could impact the microbiome and potentially *Salmonella* dynamics in cattle (53).

Despite existing research on dietary modifications, the effect on *Salmonella* in cattle remains underexplored with inconsistent results (44–46). For example, brewer’s by-products, corn gluten feed, and cottonseed hulls have been linked to increased *Salmonella* prevalence (*P* < 0.01) in feces (44). On the contrary, in other studies, brewer’s by-products and whole cottonseed or hulls addition showed no significant effect on *Salmonella* prevalence (*P* > 0.05) in feces (47, 54). This inconsistency may be attributed to various factors influenced by the dietary changes, including pH levels, volatile fatty acid concentrations, and fiber dynamics (55, 56). Alternatively, while fasting can lead to no-starch availability increasing the rumen pH, potentially increasing *Salmonella* growth (57, 58), feeding after a period of fasting can help *Salmonella* growth in the rumen (59). Therefore, another potential pre-harvest intervention is feeding management, such as preventing feeding after fasting periods.

**Fig 1:**
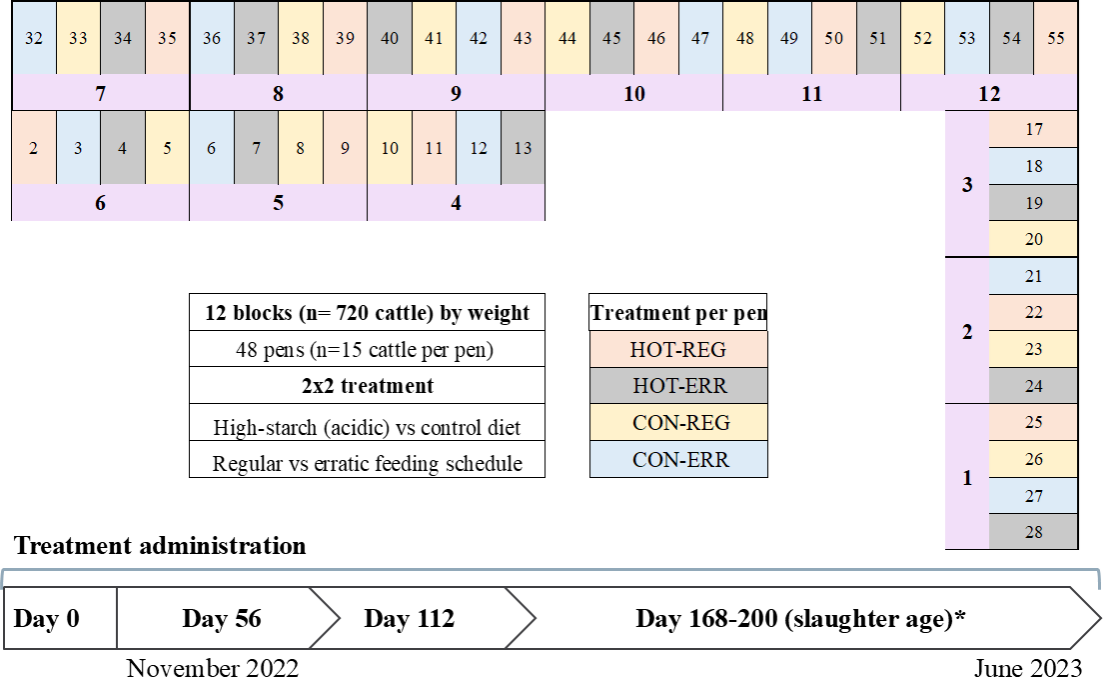
Study design (block, pen, and treatment allocations) at the feedlot and study timeline. Fecal and soil samples were collected on days 56, 112, and 168+ (harvest age). *Hide swabs were collected the day before harvest, and lymph nodes were collected during harvest. Treatment per pen is color coded as shown in the figure legend. Each block is represented bellow the respective pens in purple.

To reduce the food safety risk associated with *Salmonella* in final beef products, there is a clear need for studies exploring alternative pre-harvest interventions targeting *Salmonella* in cattle and potentially their environment. In this study, our objective was to investigate *Salmonella* populations in cattle enrolled in a randomized, longitudinal, controlled feedlot trial that was previously designed to evaluate the impact of diet (high-starch vs low-starch) and feeding management practices (regular vs. erratic) on liver abscess formation and cattle performance (Fig. 1).

## RESULTS

### Descriptive statistics

A total of 863 fecal samples, 134 lymph nodes, 309 hide swabs, and 288 composite soil samples were collected (Table 1). From these samples, a total of 653 *Salmonella* isolates were recovered. Overall, fecal, hide, lymph node, and composite pen-soil *Salmonella* prevalence estimates (95% *CIs*) were 0.33 (0.30–0.36), 0.55 (0.49–0.61), 0.26 (0.19–0.36), and 0.59 (0.54–0.65), respectively. *Salmonella* prevalence estimates (95% *CIs*) in feces were 0.22 (0.17–0.27), 0.23 (0.18–0.29), and 0.51 (0.45–0.57) for days 56, 112, and 168+, respectively. Composite soil *Salmonella* prevalence (95% *CIs*) for the side and center area of the pen, respectively, were 0.42 (0.28–0.57) and 0.46 (0.31–0.60) for day 56, 0.44 (0.29–0.59) and 0.50 (0.35–0.64) for day 112, and 0.83 (0.70–0.92) and 0.89 (0.77–0.96) on day 168+.

**TABLE 1:** Observed *Salmonella* prevalence and 95% *CIs* across sample types and days

Sample type	Day	# Samples	# Positive	*Salmonella* prevalence and 95% *CIs*
Fecal	Overall	863	278	0.33 (0.30–0.36)
	Day 56	286	58	0.22 (0.17–0.27)
	Day 112	268	63	0.23 (0.18–0.29)
	Day 168+	309	157	0.51 (0.45–0.57)
Composite soil (side or center)	Overall	288	170	0.59 (0.54–0.65)
	Day 56—side	48	20	0.42 (0.28–0.57)
	Day 56—center	48	22	0.46 (0.31–0.60)
	Day 112—side	48	21	0.44 (0.29–0.59)
	Day 112—center	48	24	0.50 (0.35–0.64)
	Day 168+—side	48	40	0.83 (0.70–0.92)
	Day 168+—center	48	43	0.89 (0.77–0.96)
Hide swab	Day 168+	309	170	0.55 (0.49–0.61)
Lymph node	Day 168+	134	35	0.26 (0.19–0.36)

### Phenotypic antimicrobial susceptibility profile

Of the 653 total isolates recovered, 536 *Salmonella* isolates from samples collected on days 56 and 168+ were tested for phenotypic antibiotic susceptibility. Of those, 525 (97.9%) were found to be pansusceptible. Seven isolates were resistant to at least one antibiotic, and among these, four isolates showed intermediate resistance. Whereas four isolates exhibited only intermediate resistance to at least one antibiotic.

Among the seven isolates that were resistant to at least one antibiotic tested, one isolate recovered from soil on day 56 that was resistant to ampicillin, chloramphenicol, and tetracycline was classified as multidrug-resistant (MDR, defined as resistant to ≥3 antibiotic classes). Another soil-origin isolate, also from day 56, was resistant to chloramphenicol and tetracycline. These soil isolates were recovered from separate pens that were not adjacent to each other. In addition, there were five fecal origin isolates from different days that were resistant only to ampicillin, with four of them also displaying intermediate resistance to either amoxicillin (*n* = 3) or chloramphenicol (*n* = 1). Among the isolates exhibiting only intermediate resistance, two were from hides and one from a lymph node, all showing intermediate resistance to cefoxitin, while one additional fecal isolate from day 56 displayed intermediate resistance to chloramphenicol. No resistance was observed against azithromycin, ceftriaxone, ciprofloxacin, gentamicin, meropenem, nalidixic acid, sulfisoxazole, trimethoprim/sulfamethoxazole, and colistin. All tested isolates were classified as intermediate for colistin due to the lack of CLSI-established breakpoints for susceptible classification. The distribution of isolates among the tested antibiotic concentrations, along with the associated CLSI and NARMS breakpoints used for classification, is presented in Table 2.

**TABLE 2:** Percentage distribution of MIC values for 536 *Salmonella* isolates tested against 14 antibiotics^*c*^^*,^d^,^e^*^

Antibiotic	Exact (Clopper-Pearson)		Minimum inhibitory concentration MIC (μg/mL) distribution and classification (%) (*n* = 536)															
	R (%)	95%*CI*	0.015	0.03	0.06	0.12	0.25	0.5	1	2	4	8	16	32	64	128	256	512
Amoxicillin/ clavulanic acid^*a*^	0.00	0.000–0.006							92.91	5.6	0.37	0.56	0.56	**0.00**				
Ampicillin	1.12	0.004–0.024							87.13	11.57	0.19	0.00	0.00	**0.00**	**1.12**			
Azithromycin^*b*^	0.00	0.000–0.006					0.00	0.00	0.00	20.52	76.12	3.36	0.00	**0.00**	**0.00**			
Cefoxitin	0.00	0.000–0.006							0.00	29.29	64.74	5.41	0.56	**0.00**				
Ceftriaxone	0.00	0.000–0.006					99.81	0.19	0.00	0.00	**0.00**	**0.00**	**0.00**	**0.00**	**0.00**			
Chloramphenicol	0.37	0.000–0.013								0.00	20.9	78.36	0.37	**0.00**	**0.37**			
Ciprofloxacin	0.00	0.000–0.006	66.79	33.21	0.00	0.00	0.00	0.00	**0.00**	**0.00**	**0.00**							
Gentamicin	0.00	0.000–0.006					34.14	62.31	3.54	0.00	0.00	0.00	**0.00**					
Meropenem	0.00	0.000–0.006			99.44	0.37	0.19	0.00	0.00	0.00	**0.00**							
Nalidixic acid	0.00	0.000–0.006						0.00	0.00	13.99	85.26	0.75	0.00	**0.00**				
Sulfisoxazole	0.37	0.000–0.013											45.9	43.47	9.89	0.37	0.00	**0.37**
Tetracycline	0.37	0.000–0.013									99.63	0.00	**0.00**	**0.00**	**0.37**			
Trimethoprim/ sulfamethoxazole^*a*^	0.00	0.000–0.006				99.81	0.19	0.00	0.00	0.00	**0.00**							
Colistin	0.00	0.000–0.006					88.43	11.19	0.37	0.00	**0.00**	**0.00**						

^*a*^ The MIC of the first antibiotic in a combination is listed.

^*b*^ NARMS breakpoints were used for classification.

^*c*^ R: Resistance. CI: confidence intervals 95% using exact Clopper-Pearson.

^*d*^ A one-sided 97.5% confidence interval was used when the prevalence estimate was zero.

^*e*^ Grey fields represent a concentration not tested for that particular antibiotic. White fields represent a concentration tested. Bolded values represent resistant percentage. Intermediate classification range is indicated with vertical bars.The numbers in grey areas are right-censored MICs. Colistin has no susceptible category, according to CLSI M100.

### Multi-level logistic regression model

**Fig 2:**
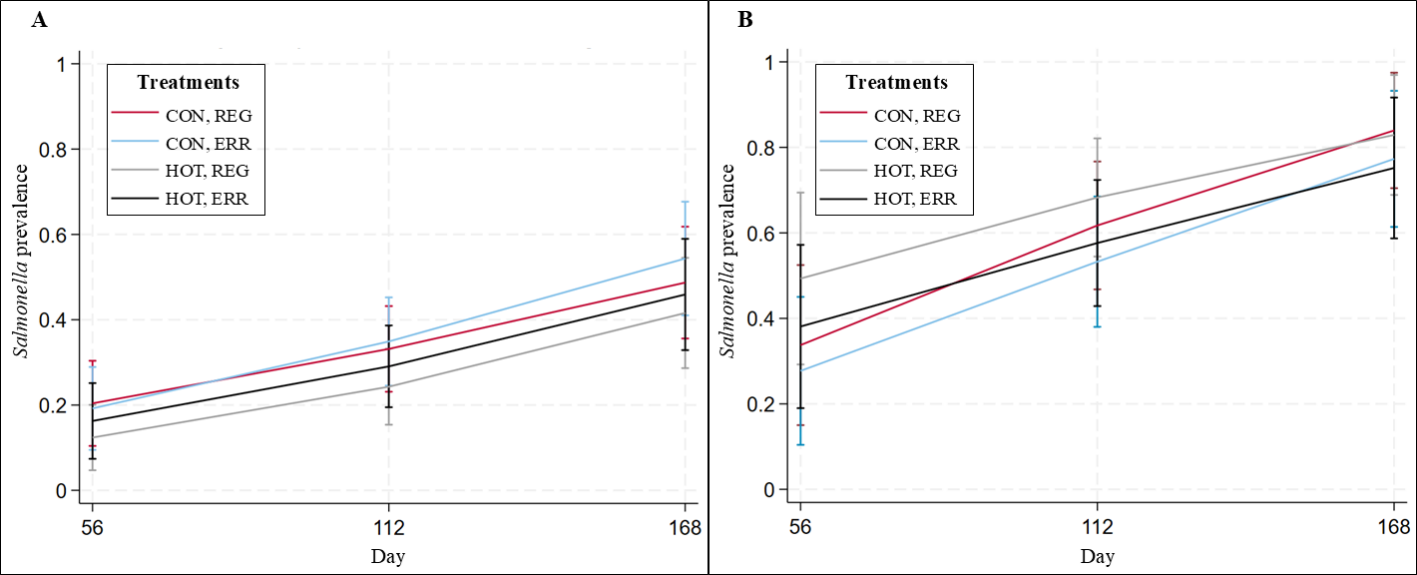
Marginal predicted fecal (**A**) and composite soil (**B**) *Salmonella* prevalence and 95% *CIs* by day and treatment.

**Fig 3:**
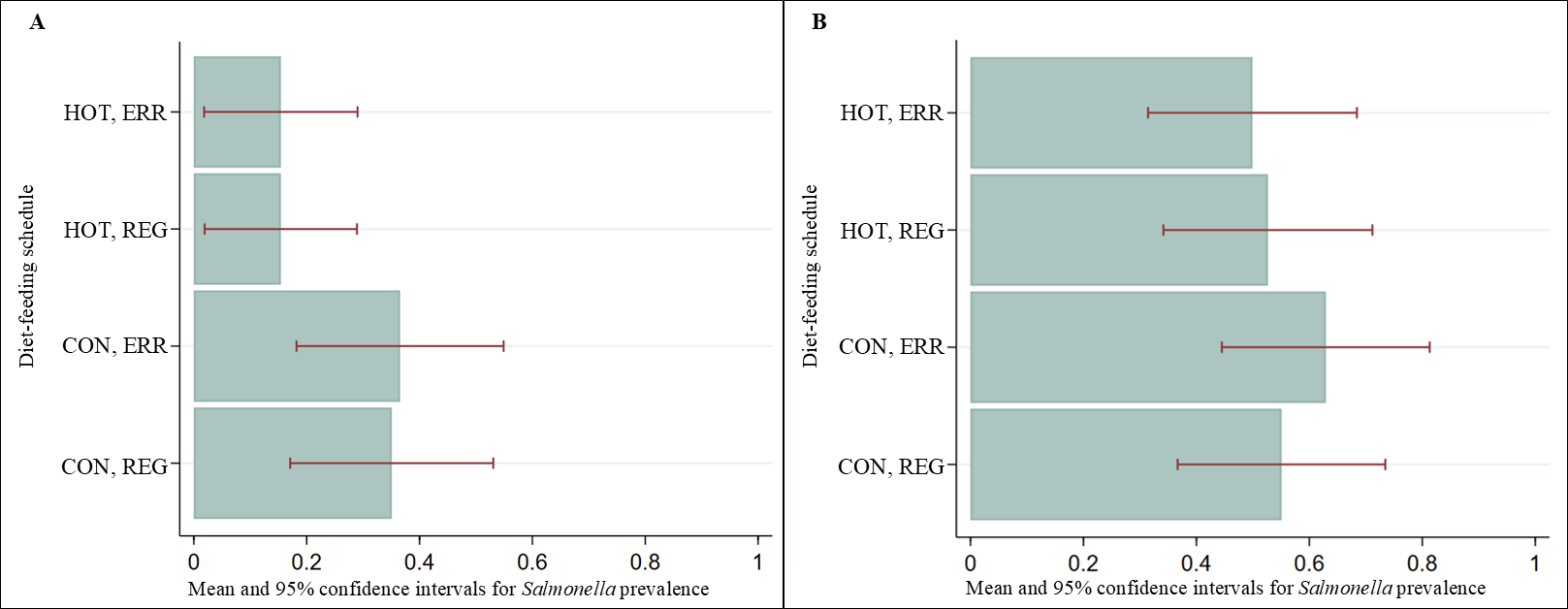
Marginal predicted lymph node (**A**) and hide (**B**) *Salmonella* prevalence and 95% *CIs* by treatment.

The marginal predicted *Salmonella* prevalence estimates in cattle feces were least across all days in cattle fed the HOT diet and greatest in cattle fed the CON diet, regardless of the feeding management regimen (Fig. 2A). Day influenced overall *Salmonella* prevalence (*P* < 0.001) in feces as summer approached, increasing from 0.20 (95% *CIs* 0.15–0.25) on day 56 to 0.50 (95% *CIs* 0.45–0.56) on day 168+ (Fig. 2A). Similar patterns were observed for *Salmonella* prevalence in composite soil samples, with the effect of day (*P* = 0.001) increasing prevalence in both side (*P* = 0.046) and center (*P* = 0.008) of the pen samples from 0.44 (95% *CIs* 0.33–0.54) on day 56 to 0.86 (95% *CIs* 0.79-0.93) on day 168+ (Fig. 2B). *Salmonella* lymph node prevalence was less in the cattle fed the HOT diet for both ERR (0.15 [95% *CIs* 0.02–0.29]) and REG (0.15 [95% *CIs* 0.02–0.29]) compared to cattle that were fed the CON diet with ERR (0.37 [95% *CIs* 0.18–0.55]) and REG (0.35 [95% *CIs* 0.17–0.53]) feeding management (Fig. 3A). The effects of diet (*P* = 0.1043) and feeding management (*P* = 0.9107) were not significant on *Salmonella* prevalence in lymph nodes; however, the HOT diet reduced *Salmonella* prevalence by 0.20 (95% *CIs* −0.02 to 0.43). Results were similar for soil (Fig. 2B) and hide swabs (Fig. 3B).

## DISCUSSION

Our study is the first to explore the relationship between a high-starch (HOT) diet, erratic (ERR) feeding management, and *Salmonella* dynamics in cattle. Although not originally designed for this purpose, the data provided preliminary insight into the connection between *Salmonella* and the dietary treatments evaluated.

In this study, we reported a *Salmonella* prevalence of 0.83 (95% *CIs* 0.70–0.92)–0.89 (95% *CIs* 0.77–0.96) in composite soil samples from day 168+, nearly double the levels observed in soil collected during winter on days 56 and 112 as the study progressed into summer on day 168+. This finding aligns with previous *Salmonella* studies conducted at the same feedlot, especially during the summer months (16, 60, 61). Similarly, Nickodem et al., 2023, reported a 76.0% (146/192) prevalence in the same feedlot environment (16). Even though sampling day affected fecal and soil *Salmonella* prevalence (*P* < 0.05) (Fig. 2), this likely reflects seasonal and ambient temperature effects in the Panhandle region, with lower prevalence observed during winter compared to warmer months (35, 60). This was an expected outcome because *Salmonella* is reported to grow within a temperature range of 5°C–46°C, with optimal growth occurring between 35°C and 37°C (62, 63). However, during our visits to the feedlot, we recovered viable *Salmonella* from soil at temperatures as low as −10°C. The survival of *Salmonella* at temperatures below 0°C may be attributed to variations in serotypes. For example, *Salmonella* Anatum has been reported to show better adaptation to lower temperatures compared to other serotypes (62).

Research indicates that *Salmonella* is affected by the pH changes in the rumen and abomasum, as diets that increase volatile fatty acids (VFAs) concentrations can lower the rumen environmental pH, leading to a reduction in *Salmonella* prevalence *in vitro* (46, 58). Although this study did not report pH values observed in the cattle GIT, Schneid et al. (unpublished data) documented a significant reduction in both ruminal and fecal pH (*P* < 0.01) in cattle fed the HOT diet. Under typical ruminal conditions, pH can vary between 5.5 and 6.0 for cattle consuming a high-grain diet and 6.0 and 7.0 for a high-roughage diet (64). For *Salmonella* to be shed in the feces, it must pass the low ruminal pH (pH <7.0) and then survive in the lower pH (pH 2.1) of the abomasum (65). Although *Salmonella* growth is enhanced in neutral pH environments (pH = 7.0), studies reported that *Salmonella* Typhimurium can adapt to survive in lower pH environments (pH 3.0–4.0) through an adaptive mechanism known as the acid tolerance response (66–68). This adaptation occurs after brief exposure to moderately low pH levels of 5.5–6.0 (69). Even though this complexity makes it difficult to recommend a single dietary approach for *Salmonella* reduction, there is also a gap in the literature about the acid adaptation ability of other *Salmonella* serotypes.

Our results did not show a statistically significant treatment effect of diet or feeding management on *Salmonella* prevalence across the different sample types and days evaluated during the feeding period and at harvest. Nevertheless, the HOT diet notably reduced *Salmonella* prevalence in lymph nodes (Fig. 3A) and moderately in feces (Fig. 2A). This outcome is not surprising, as high-starch diets are reported to lower ruminal pH (46, 58), which could potentially reduce *Salmonella* prevalence in the GIT and subsequently, in lymph nodes. Given that *Salmonella* can migrate from the GIT to lymph nodes through Peyer’s patches, become sequestered in macrophages, translocated to the lymph nodes via the lymphatic system (70), and considering that *Salmonella* in cattle lymph nodes and feces is highly clonal (36), this connection is plausible.

Our findings suggest that feeding cattle with a HOT diet may help mitigate *Salmonella* in beef products, especially ground beef, which is prone to contamination from cattle lymph nodes. Such contamination poses a significant financial burden for the beef industry due to costly recalls of contaminated beef products linked to human outbreaks. For example, the 2018 *Salmonella* Newport outbreak led to the recall of 12.1 million pounds of ground beef (13). However, it is important to note that the original study had a limited number of cattle per pen, which is not reflective of commercial settings with larger numbers of cattle per pen. Also, it was designed to measure the effects of treatments on liver abscesses and animal performance. Since the diets were fed to the cattle throughout the study and sampling was carried out at only three periods of time (days 56, 112, and 168+) from only a subset of the cattle (three out of 15 per pen), we were not able to evaluate the effect of these diets on *Salmonella* prevalence in cattle lymph nodes prior to day 168+. Consequently, while we observed a notable reduction in *Salmonella* prevalence in cattle fed a HOT diet, the results were not statistically significant due to the limited sample size. On the other hand, we collected fecal samples as early as day 56, and, on that day, we observed a slightly lower prevalence in cattle fed with HOT compared to CON. *Salmonella* prevalence remains unknown in the early days of the study or the feeding period. This could be an important outcome for our study since the dietary effects on *Salmonella* prevalence could be observed as short as 7 days (71).

For example, other studies evaluating dietary pre-harvest interventions have shown the importance of the interval between sample collection and treatment administration time in the observed effect on *Salmonella* prevalence (47, 71). While Fossler et al. 2005 observed no effect of whole cottonseed or hulls in the diet on *Salmonella* prevalence in feces collected with an interval of 68 days on average, Dargatz et al. (71) reported an increased *Salmonella* prevalence in feces after 7 days of cottonseed hulls included in the diet. Therefore, additional research is necessary to determine the effects of high-starch diets at varying concentrations and durations before harvest. If effective, this approach could serve as a pre-harvest intervention, supporting a “One Health” strategy that mitigates public health risks from *Salmonella* while also increasing efficiency in weight gain. However, findings by Schneid et al. (72) indicated a 53% increase in liver abscesses among cattle fed a HOT diet, raising concerns about its long-term suitability for animal health (72). Therefore, performance and liver abscess frequency are key aspects that must be evaluated within the framework of such research, since lower performance would not be an incentive for feedlot producers to apply these interventions. While *Salmonella* in liver abscesses is not a public health concern, it is of animal health importance because of the potential synergism between *Salmonella* and *F. necrophorum* in the formation of liver abscesses in cattle (73).

In our study, nearly all *Salmonella* isolates were pansusceptible to 14 antibiotics, except for two isolates found in the feedlot environment that exhibited phenotypic resistance to ampicillin (a beta-lactam), and/or tetracycline, and chloramphenicol (an amphenicol), and five isolates that were resistant to only ampicillin. It is also important to note that standard bacterial isolation techniques may introduce potential bias, as bacteria exposed to various stresses, such as acidic or cold environments can become viable but non-culturable. These conditions may prevent successful cultivation even when temperature and nutrient stimuli are applied (74). In addition, the use of selective media like Rappaport-Vassiliadis can influence serotype recovery (75), potentially impacting observed phenotypic AMR profiles, because certain resistant patterns are highly associated with specific serotypes (76, 77).

We were not able to determine a clear pattern between the two resistant isolates because they were collected from different areas (side and center of the pen) of non-adjacent pens on day 56 only. Even though we isolated *Salmonella* from 108 samples from day 112, they were not evaluated for MIC because our focus was to compare the early information of the study with the time of harvest. Therefore, information on the resistance patterns expressed in isolates on day 112 is nonexistent, which limits the inferences about the remaining resistance patterns throughout the study. Moreover, the feedlot environment, water, and feed can be contaminated by wildlife activity (78) or dust (79). Vectors such as horn flies can also transmit *Salmonella* during blood feeding (80, 81). It is unclear whether the cattle initially carried these resistant isolates and contaminated the environment, if they acquired them from the environment, or if the soil contamination resulted from external factors. Consequently, the environmental influence on *Salmonella* dynamics from the start of treatment administration to harvest remains unknown.

While these resistant strains were only detected in the environment, prior research in the same feedlot has demonstrated that *Salmonella* from cattle fecal and lymph node samples is highly clonal (36), suggesting that these strains could also be present in the cattle. Although these antibiotics are not used for treating *Salmonella* infections in humans, they are still considered highly important for human medicine (iris.who.int/bitstream/handle/10665/312266/9789241515528-eng.pdf?sequence=1 [Accessed June 2025]) (82). Because these antibiotic classes are also approved for use in cattle for disease management (www.fda.gov/animal-veterinary/antimicrobial-resistance/2022-summary-report-antimicrobials-sold-or-distributed-use-food-producing-animals [Accessed June 2025]) (83), their use during the study could result in selection pressure, potentially leading to the persistence of these strains in cattle and the feedlot environment. This notion is also supported by two studies conducted in the same feedlot (60, 61). Ohta et al. (61) observed that treatment with chlortetracycline (a tetracycline) and ceftiofur (a third-generation cephalosporin) increased (*P* < 0.05) MDR *Salmonella* in feces, indicating antibiotic-driven selective pressure on an existing resistant population (61). By contrast, Levent et al. (60) reported no effect of ceftiofur or tulathromycin (a macrolide) on *Salmonella* prevalence and MDR in cattle feces, hide, or lymph nodes, likely due to the absence of pre-existing resistant strains (60).

Changes in the cattle GIT microbiome are predominantly attributed to diet, as the physical and chemical characteristics of the feed determine the availability of different niches (53). Therefore, a diet that alters the pH can also impact the resistome in cattle. Antibiotic-resistant *Salmonella* in cattle and the feedlot environment is highly clone-specific (60, 61), and dietary changes can create selection pressure for certain serotypes. For example, research has shown that a pH greater than 8 can inhibit the growth of *Salmonella* serotype Typhimurium while favoring Anatum and Newport (58). This potential serotype selection due to dietary changes and consequently pH alteration can also influence the AMR profiles observed in *Salmonella* in cattle. Future research is needed to explore the potential effects of dietary changes on the serotype and clonal distribution of *Salmonella* in cattle and their environment.

## MATERIALS AND METHODS

### Study design

A longitudinal double-blinded randomized controlled feedlot study as an acidosis challenge model to induce liver abscess was conducted by (72, 72) at the West Texas A&M University (WTAMU) Research Feedlot in Canyon, Texas, USA. For this study, 744 crossbred cattle were acquired from a backgrounding operation in central Texas and transported to the feedlot. Previously published cattle-receiving management was implemented (72), and on day −1a total of 720 cattle were weight-blocked into 12 blocks with 4 pens each and 15 cattle per pen. All animals were randomized into soil-surfaced treatment pens (*n* = 48), and within each block, pens received one of four diet-feeding management regimens as a part of the 2 × 2 factorial arrangement of treatments including diet with 64.4% (HOT) or 49.1% (CON) total starch with either erratic feeding management (ERR) or consistent daily feeding management (REG) for 168–222 days until harvest (referred to as 168+) (Fig. 1).

Major components of the diet included steam-flaked corn, sweet bran, soybean meal, corn stalks, molasses blend, growing supplement, and finishing supplement with monensin (Rumensin, Elanco, Greenfield, IN, USA) throughout the study period. CON diet resembles recommendations of feedlot nutritionists (84), while HOT diet has a higher concentration of readily fermentable starch, low roughage, and no grain-milling co-products. ERR feeding management was defined as random changes in feed quantity (85% followed by 115% of the previous 4-day average once per week) and feed delivery time (delayed 1, 2, 3, or 4 hours twice a week). Whereas consistent feeding management involved a consistent feed to appetite quantity (<2.27 kg feed carryover) and feed delivery time (within 15 min of the previous day). More details about the study design and treatments (ingredient and nutrient composition of diets) were previously published by (72, 72).

### Sample collection and storage

Fecal and soil samples were collected during the feeding period (days 56 and 112), and on the day before harvest (day 168+), whereas the subiliac lymph nodes and hide swabs were collected only at harvest or the day before harvest. This study was conducted starting in November 2022 and ending in June 2023 (Fig. 1). All live animal procedures were approved by the WTAMU Institutional Animal Care and Use Committee (IACUC; approval number: 2022.07.002) (72).

For fecal samples, a randomly selected subset (*n* = 6) of cattle was sampled via rectal palpation using obstetric gloves per animal. In addition, two pen composite soil samples—from the side and center of each pen—were collected from each pen using plastic disposable spoons on days 56, 112, and the day before harvest. Soil samples were taken from five different spots every 5 meters along the side transect of the shared border between two pens, as well as from five spots in the center of each pen. The day before harvest, a 1 m^2^ area of the brisket hide area of individual cattle was swabbed using a sterile sponge swab (VWR, Radnor, PA, USA) pre-suspended in 25 mL 1× phosphate-buffered saline pH 7.4 (PBS, Thermo Fisher Scientific, Waltham, MA, USA). One subiliac lymph node per animal was collected from three cattle per pen during harvest. After each collection, samples were transported to the School of Veterinary Medicine at Texas Tech University (Amarillo, Texas), the laboratory for storage and processing.

Upon arrival at the laboratory, all samples were vortexed, homogenized, and stored in a 1:1 ratio in sterile 50% glycerol and without glycerol at −80°C for further analyses. For the lymph nodes, samples were pre-enriched and stored under the same conditions.

### *Salmonella* isolation

*Salmonella* was isolated using modified previously published methods (60, 61, 85, 86). Briefly, fecal, composite soil samples, and hide swab samples were homogenized by thoroughly vortexing. Then a 0.5 g aliquot of the stored sample was suspended in 5 mL of tryptic soy broth (TSB, Difco, BD, Franklin Lakes, NJ, USA) and homogenized by vortexing for 10 seconds. Individual lymph nodes were placed in a sterile petri dish, and then the surrounding fascia and fat were trimmed aseptically using sterile scissors. The trimmed lymph nodes were surface sterilized for two to three seconds in boiling water, placed in sterile plastic stomacher filter bags (Fisherbrand, Thermo Fisher Scientific), and pulverized with a rubber mallet (85). Then, 80 mL of TSB was added to the bags containing the pulverized lymph nodes, and the suspensions were homogenized at 230 rpm for 30 seconds using a stomacher (Circulator 400; Seward, West Sussex, UK).

Later, all TSB suspensions were incubated at 42°C for 3 hours. Following initial incubation, 1 mL of suspension was transferred into 9 mL of tetrathionate broth (TTB, Difco, BD) containing 200 µL iodine solution (Remel, Lenexa, KS, USA) and incubated at 37°C for 18–24 hours. After incubation, 100 µL of the bacterial suspension was transferred into 10 mL Rappaport-Vassiliadis R10 broth (RV, Difco, BD) and incubated at 42°C for 18–24 hours. After incubation, a 50 µL aliquot was spiral plated onto brilliant green agar (BGA, Difco, BD) using the Eddy Jet 2W spiral plater (Neutec Group Inc., Farmingdale, NY, USA). The plates were further incubated at 37°C for 18 hours. After incubation, *Salmonella*-like colonies were streaked on tryptic soy agar with 5% sheep blood (Blood agar, Difco, BD) and confirmed by *Salmonella* O Antiserum Poly A-I Factors 1-16 (Difco, BD). Agglutination-positive isolates were confirmed as *Salmonella* and preserved in cryobeads at −80°C.

### Phenotypic antimicrobial susceptibility testing

The MIC of 14 antibiotics representing a total of nine classes of antibiotics, including aminoglycosides (gentamicin), beta-lactams (amoxicillin + clavulanic acid, ampicillin, cefoxitin, ceftriaxone, and meropenem), folate pathway antagonists (trimethoprim + sulfamethoxazole), macrolides (azithromycin), amphenicols (chloramphenicol), quinolones (ciprofloxacin and nalidixic acid), tetracyclines (tetracycline), and polymyxin (colistin) were determined for only the 536 isolates recovered from days 56 and 168 + using the broth microdilution method.

Briefly, purified isolates were streaked on tryptic soy agar with 5% sheep blood (Difco, BD) and incubated at 37°C overnight. Following incubation, 1.5 × 10^8^ CFU/mL were aseptically transferred to 5 mL of demineralized water using a 0.5 McFarland Standard and nephelometer (Trek Diagnostic Systems, Thermo Scientific, Oakwood Village, OH, USA). A 10 µL aliquot of the suspension was transferred to 11 mL Mueller Hinton broth (MHB, Trek Diagnostic Systems, Thermo Scientific). Finally, a 50 µL aliquot of the MHB culture was inoculated using the National Antimicrobial Resistance Monitoring System (NARMS) Gram-negative CMV5AGNF plates (Trek Diagnostic Systems, Thermo Scientific) with the Sensititre AIM Automated Inoculation Delivery System (Trek Diagnostic Systems, Thermo Scientific). *Escherichia coli* ATCC 25922, *Escherichia coli* ATCC 35218, *Pseudomonas aeruginosa* ATCC 27853, *Staphylococcus aureus* subsp. *aureus* ATCC 29213, and *Enterococcus faecalis* ATCC 29212 (American Type Culture Collection, Manassas, VA, USA) isolates were used as positive controls for each lot. Following inoculation, the plates were placed into the Sensititre ARIS HiQ system (Trek Diagnostic Systems, Thermo Scientific) and incubated at 34°C–36°C. After incubation, plates were automatically read at 18 hours to obtain MICs. Isolates were later classified as susceptible, intermediate, and resistant using the Clinical and Laboratory Standards Institute (CLSI) breakpoints where applicable (87); otherwise, NARMS breakpoints were used (www.cdc.gov/narms/about/antibiotics-tested.html?CDC_AAref_Val=https://www.cdc.gov/narms/antibiotics-tested.html [Accessed June 2025])(88).

### Statistical analysis

Statistical analyses and graphical visualizations were performed in STATA statistical software v.17.0 (StataCorp LLC, College Station, TX, USA), including descriptive and inferential statistics. Prevalence data were obtained from *Salmonella*-confirmed isolates. A multi-level logistic regression model was used to estimate treatment effects on *Salmonella* prevalence, incorporating fixed effects of sampling day and treatments including diet and feeding (CON-REG, CON-ERR, HOT-REG, and HOT-ERR), with pen and animal ID as a random effect when applicable. Predictive marginal prevalence and 95% *CIs* were obtained for each model separately for each sample type and were visualized with horizontal bar and line graphs. Resistant proportions and exact Clopper-Pearson confidence intervals were estimated. Statistical significance was declared when *P* ≤ 0.05.

## Data Availability

The data supporting the findings of this study are available from the corresponding author, Dr. Gizem Levent, upon reasonable request. Due to the sensitive nature of the research, which includes coded laboratory identifiers and information that could potentially compromise the privacy of research sponsors and collaborators, the data are not publicly accessible.
